# Dose-Response of High-Intensity Training (HIT) on Atheroprotective miRNA-126 Levels

**DOI:** 10.3389/fphys.2017.00349

**Published:** 2017-05-30

**Authors:** Boris Schmitz, Katrin Schelleckes, Johanna Nedele, Lothar Thorwesten, Andreas Klose, Malte Lenders, Michael Krüger, Eva Brand, Stefan-Martin Brand

**Affiliations:** ^1^Institute of Sports Medicine, Molecular Genetics of Cardiovascular Disease, University Hospital MuensterMuenster, Germany; ^2^Internal Medicine D, Nephrology, Hypertension and Rheumatology, University Hospital MuensterMuenster, Germany; ^3^Department of Physical Education and Sports History, University of MuensterMuenster, Germany

**Keywords:** microRNA, lactate, angiogenesis, atherosclerosis, short sprint interval (SIT)

## Abstract

**Aim:** MicroRNA-126 (miR-126) exerts beneficial effects on vascular integrity, angiogenesis, and atherosclerotic plaque stability. The purpose of this investigation was to analyze the dose-response relationship of high-intensity interval training (HIIT) on miR-126-3p and -5p levels.

**Methods:** Sixty-one moderately trained individuals (females = 31 [50.8%]; 22.0 ± 1.84 years) were consecutively recruited and allocated into three matched groups using exercise capacity. During a 4-week intervention a HIIT group performed three exercise sessions/week of 4 × 30 s at maximum speed (all-out), a progressive HIIT (proHIIT) group performed three exercise sessions/week of 4 × 30 s at maximum speed (all-out) with one extra session every week (up to 7 × 30 s) and a low-intensity training (LIT) control group performed three exercise sessions/week for 25 min <75% of maximum heart rate. Exercise miR-126-3p/-5p plasma levels were determined using capillary blood from earlobes.

**Results:** No exercise-induced increase in miR-126 levels was detected at baseline, neither in the LIT (after 25 min low-intensity running) nor the HIIT groups (after 4 min of high-intensity running). After the intervention, the LIT group presented an increase in miR-126-3p, while in the HIIT group, miR-126-3p levels were still reduced (all *p* < 0.05). An increase for both, miR-126-3p and -5p levels (all *p* < 0.05, pre- vs. during and post-exercise) was detected in the proHIIT group. Between group analysis revealed that miR-126-3p levels after LIT and proHIIT increased by 2.12 ± 2.55 and 1.24 ± 2.46 units (all *p* < 0.01), respectively, compared to HIIT (−1.05 ± 2.6 units).

**Conclusions:** LIT and proHIIT may be performed to increase individual miR-126 levels. HIIT without progression was less effective in increasing miR-126.

## Introduction

MicroRNAs (miRNAs) are short (~21 nucleotide-long) endogenous non-coding RNAs that mediate mRNA degradation and translational repression (Filipowicz et al., 2008; Huntzinger and Izaurralde, 2011). In humans, miRNAs have been established as vital regulator of almost every physiological process including development, aging and disease (Alvarez-Garcia and Miska, 2005; Sayed and Abdellatif, 2011; Jung and Suh, 2014). Following the identification of muscle-specific miRNAs such as miR-1 and miR-133 and their role in skeletal muscle development, plasticity, and regeneration (McCarthy and Esser, 2007; Simionescu-Bankston and Kumar, 2016), miRNAs have only recently been suggested as markers for individual exercise response (Zacharewicz et al., 2013; Flowers et al., 2015; Polakovičová et al., 2016). In addition, the discovery of inducible circulating miRNAs in human plasma in combination with high-sensitive detection methods have led to the concept of novel minimally invasive approaches to monitor the physiological response to different exercise regimes. Circulating plasma miRNAs are commonly preserved by association with either RNA-binding proteins or small membranous vesicles [i.e., exosomes, microvesicles (MVs)] (Arroyo et al., 2011). In this conformation, miRNAs are involved in inter-cell communication by miRNA-rich MVs shed from the plasma membrane into the extracellular environment to regulate targets in recipient cells (Zernecke et al., 2009). Significant amounts of MVs are commonly released from endothelial cells (ECs) into the plasma and thus ECs are an abundant source of miRNA-rich MVs (Deregibus et al., 2007). One of the most frequent and EC-specific miRNAs is miR-126 which is processed from intron 7 of the *EGF-like domain-containing protein 7* (*EGFL7*) gene (Wang et al., 2008). The precursor miR-126 molecule gives rise to two mature strands, miR-126-3p and miR-126-5p (Wang et al., 2008; Zernecke et al., 2009). It has been shown that endothelial microparticles contain and transfer miR-126 as a highly bioactive molecule with considerable activity to promote EC migration and proliferation (Wang et al., 2008; Zernecke et al., 2009). As such, miR-126 mimics have already been successfully used to limit atherosclerosis and increase plaque stability in atherosclerotic mouse models (Zernecke et al., 2009), which mark miR-126 as a functional biomarker for anti-atherosclerotic training interventions in humans.

It was initially assumed that only the miRNA-3p strand with weaker 5′ thermostability was actively involved in the regulation of cellular processes, while the miRNA-5p strand would be degraded without further usage (Poissonnier et al., 2014). However, a recent study by Poissonnier and coworkers provided evidence that endothelial miR-126-5p is functional and regulates leucocyte adhesion and transmigration (Poissonnier et al., 2014). In addition, the group of Weber and colleagues reported that miR-126-5p promotes endothelial proliferation and also limits atherosclerosis independent of the -3p molecule (Schober et al., 2014). With respect to these results, which suggest a strong and specific atheroprotective capacity of miR-126, the current study focused on the effect of physical exercise on circulating miR-126 levels. It is well-known that physical activity has widespread health benefits and is an important factor in primary and secondary prevention of cardiovascular disease (Myers et al., 2002; Haskell et al., 2007). High-intensity interval training (HIIT) has been identified to be efficient for increasing health-related fitness in general (Burgomaster et al., 2008; Costigan et al., 2015; Milanović et al., 2015) and in lifestyle-induced chronic diseases such as coronary artery disease, heart failure, diabetes mellitus, hypertension, obesity, and metabolic syndrome (Weston et al., 2014). However, exercise-induced miRNA-dependent processes triggering these protective effects remain only partly understood. Therefore, the aim of this study was to explore the dose-response relationship of HIIT on miR-126-3p and miR-126-5p plasma levels in humans. We hypothesized that progressive HIIT (proHIIT) would result in increased miRNA-126 levels compared to HIIT performed at constant intensity.

## Methods

### Participants

Sixty-one young healthy moderately trained female and male students of the universities' physical education department were consecutively recruited at the Institute of Sports Medicine of the University Hospital Muenster in April 2015. All investigations were performed in accordance with the declaration of Helsinki and after the approval of the Ethical Committee of the medical association Westfalen-Lippe and the Westphalian Wilhelms-University of Muenster (project-no. 2013-231-f-S, study acronym SPORTIVA). Written informed consent of participants was obtained prior to subjects' participation in the study. Participants' baseline characteristics were as follows. Female = 31 (50.8%), age = 22.0 ± 1.84 years, height = 177.0 ± 9.70 cm, mass = 70.7 ± 11.46 kg. In total 11 participants dropped out of the study, four from the HIIT group, four from the proHIIT group and three from the LIT group due to several reasons including injury unrelated to the intervention program. Due to scheduling problems at retest, post-training data of two participants of the proHIIT group was not available.

### Randomization procedure

Participants were randomized to one of the three training groups using exercise capacity as primary parameter determined by a standardized incremental continuous running test (ICRT, as maximum performance test). The ICRT is a highly reproducible field test method and was performed as described elsewhere (Léger and Boucher, 1980; Berthoin et al., 1994; McGehee et al., 2005) with modifications (Schmitz et al., 2017). The test was performed indoors on a synthetic 200 m running track at ambient temperature (20–24°C, ~60 m above sea level) in groups of 4–6 individuals. Subjects were fitted with HR monitors combined with a wireless receiver module (Polar Team^2^, Polar Electro Oy, Kempele, Finland). The test started at 2.22 m·s^−1^, increasing by 0.56 m·s^−1^ every 3 min until total exhaustion of the participant. The pace was indicated by an automated acoustic device. Blood was sampled from participants' earlobes (20 μl heparinized capillary) for blood lactate concentration measurements (automated on analyzer Biosen S-line, EKF Diagnostics, Magdeburg, Germany) after each interval (3 min). Exercise capacity at individual lactate threshold (baseline lactate + 1.5 mmol·L^−1^; Roecker et al., 1998; Dickhuth et al., 1999) was calculated using Winlactat software version 5.0.0.54 (Mesics, Münster, Germany).

### Training and testing

#### Training interventions

The study included three different groups with a 4-week training intervention performing three exercise sessions/week (1 day off between sessions).

1. High-intensity interval training (HIIT): HIIT participants were instructed to run at maximum speed for 4 × 30 s (all-out) with 30 s of active recovery periods at warm-up speed between bouts.
2. Progressive high-intensity interval training (proHIIT): proHIIT participants started at 4 × 30 s (all-out), increasing by one extra interval each week to a final set of 7 × 30 s (all-out) with 30 s of active recovery periods at warm-up speed between bouts.
3. Low-intensity training (LIT, control group): The LIT group was used as control group and performed a continuous run at 74.75% of HR_max_ (147 ± 12 b·min^−1^) for 25 min.

All participants were students of the physical education department and had free access to the universities' athletic facilities during the intervention. Training was unsupervised and participants documented exercise sessions in individual training logs (HIIT/proHIIT, rating of perceived exertion [RPE] on 15-grade Borg scale; Borg, 1982; LIT, HR). Participants were free to perform their three exercise sessions/week during any time of the day but had to comply to the 1 day exercise/1 day of schedule. Participants were seen and interrogated at least once a week during their regular seminars. Training logs were evaluated at the end of the intervention and revealed no difference in compliance between the three groups: HIIT, 92.2 ± 12.35% vs. proHIIT, 91.7 ± 11.28% vs. LIT, 88.7 ± 18.22%; *p* = 0.793. HIIT and proHIIT participants reported RPE at 19.1 ± 0.8. Participants were involved in different other irregular activities such as resistance training, team sports, etc. which had already been performed prior to the training intervention and were distributed equally over the three training groups. Additional training was also documented in training logs. Information on participants' medication was interrogated by questionnaire. One participant reported use of allopurinol (proHIIT), one reported use of budesonide (LIT), one reported iodine supplementation (proHIIT) and three reported use of levothyroxin (2 × proHIIT and 1 × LIT). Participants' diet was not controlled.

#### Testing

Baseline and follow-up examinations were performed at the first (1-week after the initial ICRT randomization) and after the last exercise session (described above), respectively. Blood sampling for miRNA and lactate determination in the LIT group was performed before and after a 25 min low-intensity run <75% of HR_max_ (controlled by HR monitors). HIIT and proHIIT pre- and post-exercise sampling was identical at baseline as both groups performed four high-intensity runs at maximum speed for 30 s (all-out) with 30 s of active recovery (Supplemental Figure 1). At follow-up, HIIT and proHIIT participants' blood was sampled at three different time points, before, after 4 min and after 7 min for comparison. The HIIT group stopped after 4 min (= 4 bouts of exercise) and the 7 min time point equaled 3 min of rest post-exercise (Supplemental Figure 1). The proHIIT group was sampled after 4 min during exercise (30 s of passive rest for sampling instead of 30 s of active recovery) and after the 7th (final) exercise bout. To monitor performance during high-intensity runs, running speed was continuously recorded using the SmarTracks Diagnostics remote exercise monitoring and bio-feedback system (Humotion, Münster, Germany). Blood lactate concentrations are presented in Table 1.

**Table 1:** Exercise blood lactate (LA) concentration pre- and post-intervention.

**Group**	**LA pre-intervention (mmol·L^−1^)**	**LA post-intervention (mmol·L^−1^)**
LIT	1.22 ± 0.4	1.46 ± 0.5
HIIT	10.96 ± 2.1	10.82 ± 2.1
proHIIT	10.81 ± 2.7	9.81 ± 3.1

*Data are mean ± SD. LA-values were determined after four bouts of high-intensity running in the HIIT and proHIIT group and after 25 min of low-intensity running in the LIT group*.

### miRNA extraction and quantification

Blood was sampled from participants' earlobes immediately at the testing site using a 20 μl K2 EDTA capillary (Sarstedt, Nürnbrecht, Germany) and miRNA was extracted using 750 μl peqGOLD TriFast (VWR, Darmstadt, Germany) according to the manufacturer's instruction. Wehmeier and Hilberg (2014) have shown that the expression levels of miRNAs using standard immediate venous blood sampling compared to capillary blood sampling are nearly identical. Most importantly, the applied method allows the sensitive detection of acute changes in circulating miRNA levels during and directly after exercise and prevents the bias of hemolysis. Each sample was immediately supplemented with 10 nM *Caenorhabditis elegans* cel-miR-39-3p spike-in control following manufacturer's instruction (Thermo Fisher Scientific, Darmstadt, Germany) for normalization. This approach was chosen since internal reference controls have so far not been validated and it remains uncertain which transcript/control might be affected by the performed training interventions. Spike-in controls such as cel-miR-39 have been successfully applied as reference for circulating miRNAs including miR-126 (Fichtlscherer et al., 2010; Schlosser et al., 2015), but cannot control for pre-analytical variations (sample collection, storage and/or transport; Schlosser et al., 2015). As the performed sample preparation procedure had a high level of standardization in our setting (in-field 20 μl capillary sampling with immediate preparation) pre-analytical variations were reduced to a minimum. RNase-free glycogen (70 μg/sample; VWR) was used as carrier to optimize extraction efficiency as reported (McAlexander et al., 2013). Isolated RNA was resuspended in 20 μl of nuclease-free water. Quantification of mature hsa-miR-126-3p, hsa-miR-126-5p, and cel-miR-39-3p was performed by quantitative real-time polymerase chain reaction (qRT-PCR) using TaqMan Advanced MicroRNA Reverse Transcription Kit for cDNA generation and TaqMan Advanced MicroRNA Assays (Thermo Fisher Scientific, Darmstadt, Germany) blinded to participants' parameters. The Advanced MicroRNA Assay uses 5′ adaptor ligation to mature miRNAs offering the benefit to generate cDNA for all miRNAs in a single reaction, limiting the failure of cDNA generation in the analysis. In brief, 2.5 μl of RNA solution was used for qRT-PCR reactions performed in a 384-well format in duplicates on an ABI7500 fast RT- PCR system (Life Technologies Corporation, Carlsbad, USA). Relative quantification was performed using the ΔCt method and miR-126 values were expressed as (1/ΔCt)^*^ 100 for presentation. Duplicates with a difference >2 Ct were excluded from the analysis.

### Statistical data analysis

Statistical analyses were performed using SPSS, version 22.0 (Statistical Package for Social Science, Chicago, USA) and GraphPad PRISM V5.0 software (GraphPad Software Inc., La Jolla, USA). Eleven datasets were incomplete (missing test or retest data) and were not included in the post-training analysis. Data are presented as mean ± SD or 95% confidence interval (running speed data). For miR-126 measurements, qRT-PCR data is presented in duplicates with the number of analyzed subjects for miR-126-3p given in each figure (minimal number per analysis). Differences between and within groups were determined using one-way ANOVA and Bonferroni's Multiple Comparison Test. Data were tested for normal distribution using D'Agostino-Pearson normality test (omnibus K2 test). Significance was declared at *p* < 0.05.

## Results

### miR-126 exercise levels at baseline

**Figure 1:**
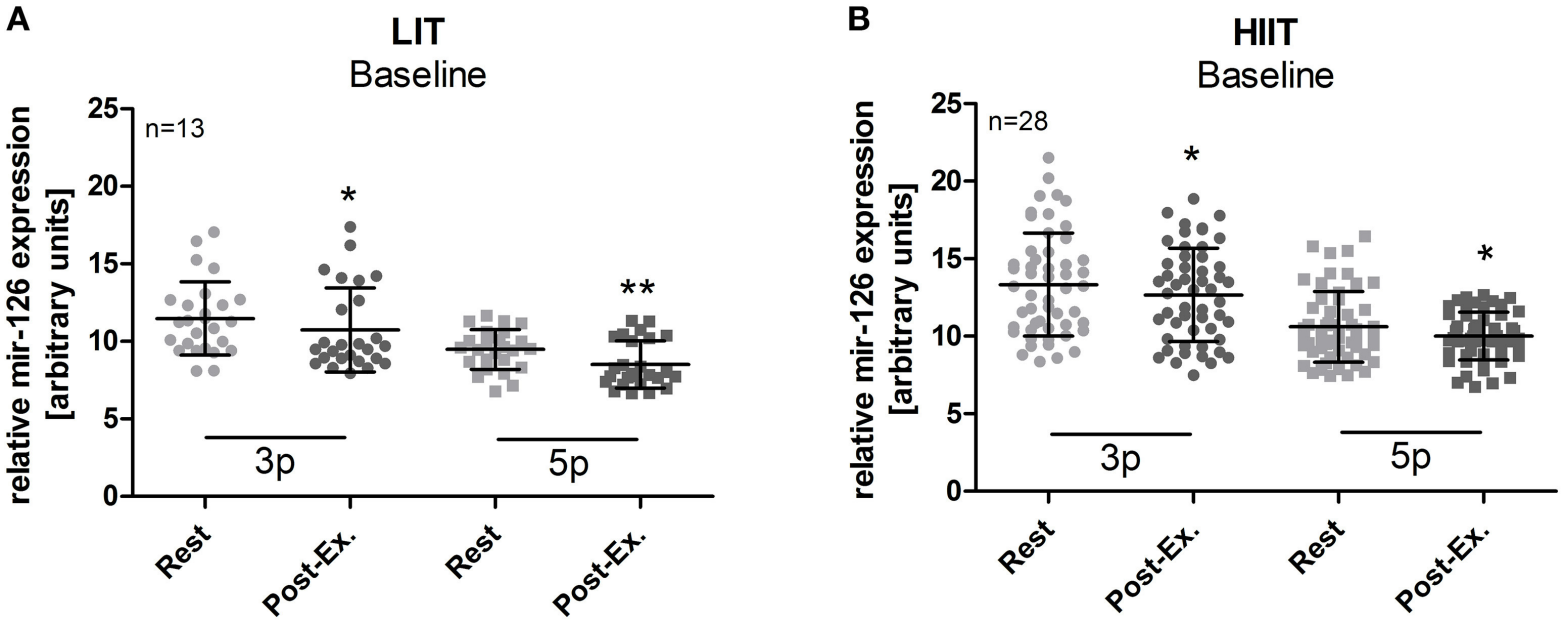
miR-126 levels were slightly reduced in response to exercise at baseline. The acute effect of a single exercise session on miR-126-3p and 5p levels before the intervention was determined in **(A)** the LIT group and **(B)** the HIIT groups. A significant decrease of miR-126-3p/-5p was detected post-exercise. LIT participants were tested before and after a 25 min low-intensity run, HIIT participants before and after 4 × 30 s (all-out) high-intensity runs with 30 s of active recovery periods at warm-up speed. Data are represented as mean ± SD. *P*-values are rest vs. post-exercise using ANOVA. ^*^p < 0.05; ^**^p < 0.01.

No exercise-induced increase in miR-126 levels was detected at baseline, neither in the LIT (after 25 min low-intensity running) nor the HIIT groups (after 4 min of high-intensity running, Figure 1). Instead, a slight but significant (all *p* < 0.05, rest vs. post-exercise) reduction for miR-126-3p and miR-126-5p was detected in all groups. No differences in miR-126 levels between male and female participants were detected (Supplemental Figure 2).

### miR-126 exercise levels at follow-up

**Figure 2:**
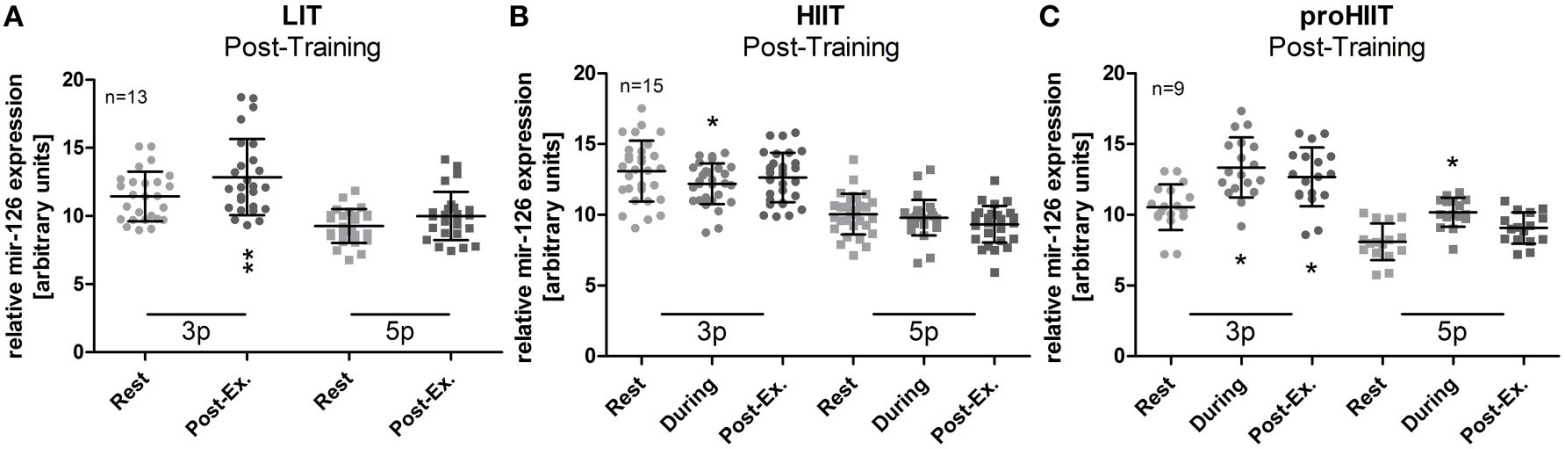
miR-126 levels were increased in response to exercise in the LIT and proHIIT group post-training. After the 4-week training intervention, a significant increase of acute miR-126 levels was detected post-exercise **(A)** in the LIT group and **(C)** in the proHIIT group (run 7), while miR-126-3p levels were still reduced in **(B)** the HIIT group (run 4). Elevated miR-126-5p levels were only detected during exercise (run 4) in the proHIIT group. miRNA levels at follow-up were analyzed directly after the last run (run 4) and after 3 min of recovery (post-exercise) in the HIIT group. In the proHIIT group, miRNA levels were analyzed during the exercise (run 4) and directly after the exercise (run 7, post-exercise). Data are represented as mean ± SD. *P*-values are rest vs. post-exercise using ANOVA. ^*^p < 0.05; ^**^p < 0.01.

**Figure 3:**
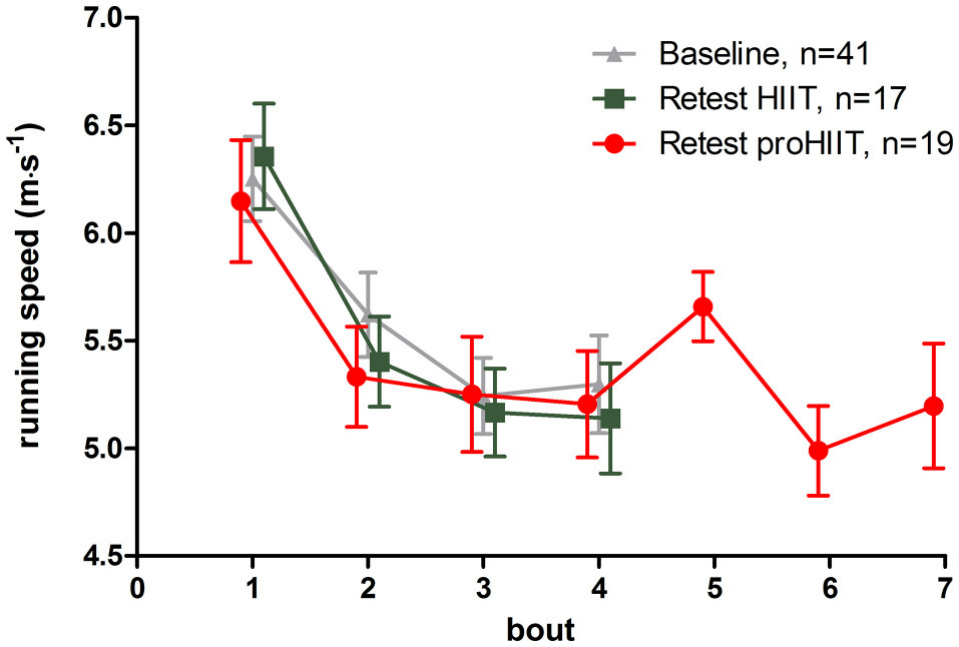
Running speed during high-intensity runs. Running speed was continuously recorded during high-intensity runs at baseline and retest to monitor exercise intensity. No significant differences in running speed at baseline and retest or between groups (bout 1–4) was detected.

After 4-weeks of training, the LIT group presented a significant (*p* < 0.01, rest vs. post-exercise) increase in miR-126-3p levels in response to the 25 min low-intensity run (Figure 2A). The concentration for miR-126-5p did not change significantly in response to exercise during follow-up (Figure 2A). In the HIIT group, miR-126-3p levels were again reduced (*p* < 0.05, rest vs. run 4) while miR-126-5p levels did not change in response to exercise during follow-up (Figure 2B). A prominent increase for both, miR-126-3p and -5p levels (*p* < 0.05, rest vs. during exercise) was seen in the proHIIT group after four bouts of exercise (i.e., 4 min, Figure 2C). For miR-126-3p, the increase remained significant (*p* < 0.05) post-exercise (Seven bouts, Figure 2C). An additional between group analysis of the follow-up data confirmed our findings. miR-126-3p levels in the LIT and proHIIT group were significantly increased in response to exercise by 2.12 ± 2.55 and 1.24 ± 2.46 units (LIT vs. HIIT, *p* < 0.001; proHIIT vs. HIIT, *p* < 0.01), respectively, compared to reduced miR-126-3p levels in the HIIT group (−1.05 ± 2.60 units). miR-126-5p levels also increased significantly in response to exercise in the LIT and proHIIT group by 1.48 ± 2.13 and 0.87 ± 1.90 units (LIT vs. HIIT, *p* < 0.01; proHIIT vs. HIIT, *p* < 0.05), respectively, compared to reduced miR-126-5p levels in the HIIT group (−0.45 ± 1.8 units). Between group analysis did not indicate any differences of the training effect on miR-126 levels between LIT and proHIIT. To control if changes in miR-126 levels depended on exercise intensity, running speed during high-intensity runs was continuously recorded. Notably, no differences in running speed existed, neither in comparison to baseline nor between HIIT and proHIIT groups at follow-up (Figure 3).

### HIIT effects on resting miR-126-3p levels

**Figure 4:**
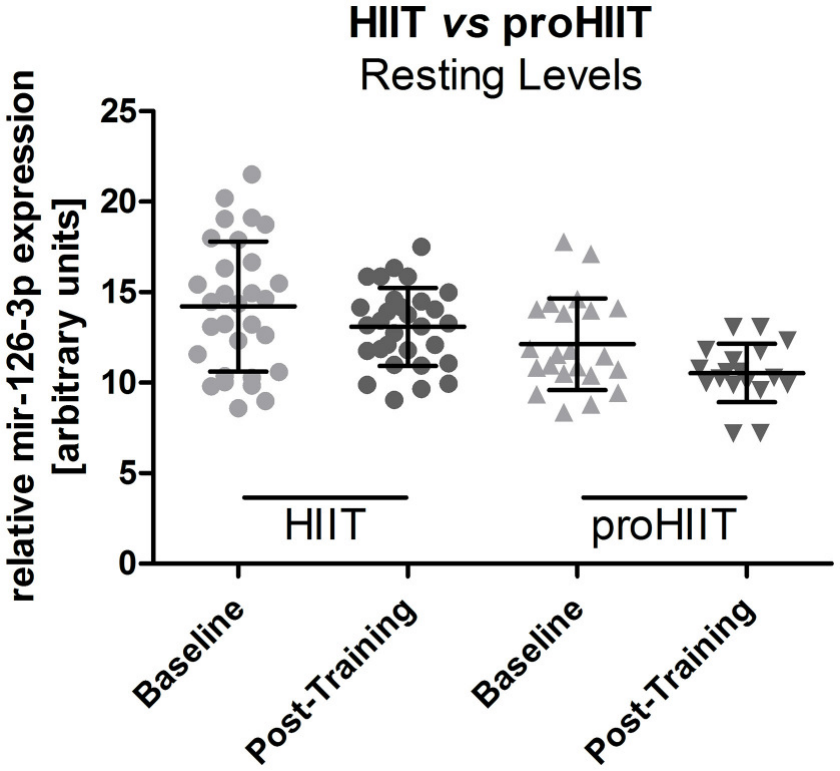
Resting miR-126-3p levels in the HIIT and proHIIT group. No significant difference between resting miR-126-3p levels was detected within each group (baseline vs. post-training; HIIT, *n* = 15; proHIIT *n* = 9). Data are represented as mean ± SD compared by ANOVA.

A within-group comparison of baseline vs. post-training miR-126 resting levels in the HIIT and proHIIT group did not reveal a lasting training effect on miR-126-3p of either HIIT protocol (Figure 4).

### Exercise blood lactate concentrations

Exercise blood lactate concentrations pre- and post-intervention for the three training groups are presented in Table 1. In the HIIT group, post-intervention lactate concentration after 3 min of passive recovery increased to 13.02 ± 5.9 mmol·L^−1^. In the proHIIT group maximum lactate concentration after seven bouts of high-intensity running was 13.86 ± 4.57 mmol·L^−1^. Incline in blood lactate concentration (rest vs. post-exercise) did not change significantly in the LIT and HIIT group, whereas it was significantly lowered in the proHIIT group post-intervention (*p* = 0.0488, Supplemental Figure 3), despite identical running speed (Figure 3).

## Discussion

In this 4-week interventional study, we analyzed the dose-response relationship of HIIT on circulating anti-atherosclerotic miR-126-3p/-5p levels in young healthy moderately trained females and males. Our main findings are: (1) A single low- or high-intensity running exercise bout at baseline did not elevate miR-126 levels, instead miR-126 levels were slightly reduced; (2) proHIIT led to increased miR-126-3p and -5p exercise levels while no elevated miR-126 levels were observed in the HIIT group; (3) the LIT group also presented increased exercise miR-126-3p levels after the intervention; and (4) both mature miR-126 forms, -3p and -5p can be elevated by regular running exercise.

While a number of studies investigated the effects of acute or prolonged exercise on miRNA abundance in tissue or plasma, only few studies have addressed changes in miRNA levels in response to specific training interventions (for review see Flowers et al., 2015; Polakovičová et al., 2016). Among other miRNAs regulated in response to physical activity, miR-126 is of particular interest as it exerts beneficial effects on vascular integrity, angiogenesis, and atherosclerotic plaque stability (Wang et al., 2008; Zernecke et al., 2009). So far, some studies focused on the effect of prolonged physical activity on miR-126 levels in rodents. The group of Oliveira reported an intensity-dependent increase of cardiac miR-126 levels after a 10-week swimming protocol imposed on female Wistar rats (Da Silva et al., 2012). The authors also documented increased capillary/fiber ratio in the heart, suggesting a relationship between the induced angiogenesis and miRNA-126 expression. By contrast, they did not observe significant changes of skeletal muscle miR-126 in male Wistar rats after the 10-week swimming protocol (Fernandes et al., 2012). However, they detected normalization of reduced skeletal muscle miRNA-126 levels in spontaneously hypertensive rats (SHR) in response to the protocol, which was paralleled by revascularization and a reduction of blood pressure (Fernandes et al., 2012). In male ApoE null C57BL/6 J mice, miR-126 levels were reported to be increased in vascular tissue after a 12-week treadmill protocol (Wu et al., 2014). In humans, Uhlemann et al. (2014) reported plasma concentrations of miR-126 after different single exercise bouts. The authors analyzed miR-126 levels after cycling in healthy individuals using a symptom-limited test and in well-trained men after cycling for 4 h at 70% of the individual anaerobic threshold as well as in trained runners after a marathon race and in trained subjects after a single resistance training. They found increased miR-126 levels in all conditions except for the resistance training, an observation in line with another report on miR-126 upregulation during prolonged aerobic exercise (Baggish et al., 2014). The group of Hilberg analyzed competitive adolescent male cyclists performing a single high-volume or high-intensity interval session reporting that the single acute high-intensity exercise did not increase miR-126 levels (Kilian et al., 2016). Taken together, these data indicate that prolonged aerobic exercise can induce elevated miR-126 levels. So far, an investigation on the effects of short intensive running exercises bouts on miR-126 is missing from the literature and most studies have not differentiated their investigations toward the two mature miR-126 forms, miR-126-3p and miR-126-5p, most likely because the anti-atherosclerotic potential of the -5p form has only recently been revealed. The group of Weber and colleagues reported that miR-126-5p affects different cellular target molecules compared to miR-126-3p and suppresses the notch1 inhibitor delta-like 1 homolog (Dlk1) inducing atheroprotective effects by promoting endothelial proliferation (Schober et al., 2014). Thus, it seems conceivable that both mature miR-126 forms act synergistically in endothelial gene expression regulation exerting cardioprotective effects.

Our data indicate that a single exercise bout in moderately trained individuals is not sufficient to induce elevated levels of miR-126 as a direct response. Instead, at the first exercise session, miR-126-3p and -5p levels were slightly reduced immediately after the exercise in all groups. This observation is partly in line with a report on moderately trained men by Radom-Aizik et al. (2010) who also observed a subsequent reduction of miR-126 levels in response to 30 min of cycle ergometry. In our study, we observed a distinct difference between the HIIT and proHIIT group after the intervention. After 4-weeks of training, the proHIIT group presented increased exercise miR-126 levels during and directly after the exercise while the HIIT group still presented slightly lowered miR-126-3p levels. Notably, exercise intensity at follow-up was not different to baseline or in comparison to the HIIT group. Since we observed a significant difference in miR-126 levels between the proHIIT and HIIT group, a dose-response effect of HIIT on miR-126 levels can be suggested.

A comparison of resting miR-126 levels between the two HIIT groups did not suggested that any of the training programs induced a permanent increase of miR-126-3p levels. Notably, when comparing the resting levels of the two HIIT groups after the training intervention we noticed a significant difference of miR-126-3p resting levels before the intervention in that the resting levels of the designated proHIIT group were significantly lower. We investigated different parameters that could explain this difference (including baseline exercise capacity, sex, age, height, activity level, medication) but detected no obvious reason for the different levels. However, it does not seem conceivable that this might have affected the diverse training response detected between HIIT and proHIIT participants as the stimulus in the proHIIT group appeared strong enough to induce higher miR-126 levels despite the lower resting levels.

The physiological model underlying exercise induced mir-126 levels might be characterized by our two main findings. mir-126 levels were not increased by the stimulus of a single exercise bout at baseline but increased quickly in only 4 min of high-intensity running after the training intervention. This rather rapid increase points to a release or secretion mechanism of miR-126 as *de novo* expression would most likely require more time (Baggish et al., 2011). With respect to the observed high reactivity it is noteworthy that the process of miRNA release into the blood stream especially in response to physical exercise is largely unknown (Chen et al., 2012; Makarova et al., 2014). However, it has been suggested that miRNA-releasing cells possess a sorting mechanism that guides specific miRNAs to enter exosomes resulting in a concentration of selected miRNAs (Zhang et al., 2015). It also seems conceivable that regular exercise training could results in modified (i.e., elevated) basal miRNA expression (Makarova et al., 2014) and thus an increased pool of concentrated miRNAs to be secreted. In particular, the molecular mechanisms involved in mammalian miR-126 expression regulation are still under debate. Pre-miR-126 is located to intron 7 of the *EGFL7* gene and promoter studies have revealed different transcription factors involved in its expression regulation including ETS1/2 (Harris et al., 2010). High shear stress has been discussed to be an activator of miR-126 expression as miR-126 is inducible by flow-sensitive Krüppel-like Factor 2 (KLF2) in zebrafish (Hergenreider et al., 2012). However, no flow or KLF2-dependent regulation of miR-126 has been detected in human ECs (Hergenreider et al., 2012). Notably, a connection between the lactate anion and miR-126 levels may be suggested based on the observation that hypoxia targets transcription factor ETS1 (Oikawa et al., 2001), while blockade of the endothelial lactate transporter MCT1 led to inhibition of hypoxia-inducible factor-dependent angiogenesis (Sonveaux et al., 2012). Preliminary *in vitro* data from our laboratories suggest that low lactate levels of ~1.5 mM have the potential to induce miR-126 expression, while lactate concentrations of 3–4 mM seem to have an inhibitory effect. Notably, blood lactate levels of our LIT group were in the potentially activating range while lactate levels in the HIIT group were quickly exceeding this range. High lactate levels were also observed in the proHIIT group after exercise, however the slope of lactate increase in this group was significantly lower after the training intervention at identical exercise intensities compared to the HIIT group. Future studies will be needed to address the potential mechanism underlying miR-126 expression regulation.

Different pathophysiological conditions have been associated with decreased miR-126 plasma levels including type 2 diabetes mellitus (Zampetaki et al., 2010). Vice versa, increased levels of miR-126 have been associated with a lower rate of cardiovascular events in patients with stable coronary artery disease (Jansen et al., 2014). HIIT, in particular, has been suggested to improve skeletal muscle insulin sensitivity (Eskelinen et al., 2015) and to increase health-related fitness in general (Burgomaster et al., 2008; Costigan et al., 2015; Milanović et al., 2015). While HIIT has also been proposed for patients with lifestyle-induced chronic diseases such as coronary artery disease, heart failure, diabetes mellitus, hypertension, obesity, and metabolic syndrome (Weston et al., 2014), it may be even detrimental for cardiac insulin sensitivity and blood flow capacity as shown for healthy but untrained middle-aged men (Eskelinen et al., 2016). It will therefore be of interest to investigate the benefits and limitations of different HIIT forms (running or cycling, progressive vs. no increment) in the general population and different patient groups in future studies. If cardioprotective effects of HIIT in healthy subjects (primary protection) and patients with different cardiovascular disease manifestations (secondary protection) can be substantiated, miR-126 could be a useful marker to control and optimize individual training interventions.

## Conclusions

We conclude that LIT and proHIIT may be performed to increase plasma levels of miR-126 in healthy females and males. Standard HIIT performed without progression may be less effective to increase miR-126 levels. The value of miR-126 as a marker to optimize individual cardioprotective training will be the scope of future studies.

## Limitations

Our results may not be directly translated to other groups or populations as our results were detected in a population of young healthy female and male Caucasians. The presented results are based on the determination of circulating miRNAs from blood. As miR-126 in particular can be highly concentrated in MVs with the potential to shuttle into target cells with high efficiency, future studies are needed to investigate the effect of physical exercise on miRNA concentrations in MVs. Even if the applied novel method for miRNA determination in the field allows for an efficient screening of a larger number of individuals, the sample size of our three training groups might be some limitation and future studies involving larger groups and longer observation periods post-exercise may generate additional insight. Our participants were free in their daily diet. Although training was not controlled, our study involved highly motivated university students with high training compliance (>90%). However, we cannot completely exclude effects of extra non-prescribed training. In addition, the extent to which participants complied with the intensity of the intervention was not measured and therefore quantification of the exercise dose was not performed.

## Author contributions

BS designed and coordinated the study, recruited and tested participants, analyzed the data, and drafted the manuscript. KS and JN performed sample preparation and miRNA measurements and analyzed data. LT tested participants and analyzed training and testing data. AK coordinated the study, recruited, and tested participants. ML participated in data analysis. MK, SB, and EB helped finalizing the manuscript. All authors read and approved the final version of the manuscript.

### Conflict of interest statement

The authors declare that the research was conducted in the absence of any commercial or financial relationships that could be construed as a potential conflict of interest.

## References

[B1] Alvarez-Garcia I. Miska E. A. (2005). MicroRNA functions in animal development and human disease. Development 132, 4653–4662. 10.1242/dev.02073 16224045

[B2] Arroyo J. D. Chevillet J. R. Kroh E. M. Ruf I. K. Pritchard C. C. Gibson D. F.. (2011). Argonaute2 complexes carry a population of circulating microRNAs independent of vesicles in human plasma. Proc. Natl. Acad. Sci. U.S.A. 108, 5003–5008. 10.1073/pnas.1019055108 21383194 PMC3064324

[B3] Baggish A. L. Hale A. Weiner R. B. Lewis G. D. Systrom D. Wang F.. (2011). Dynamic regulation of circulating microRNA during acute exhaustive exercise and sustained aerobic exercise training. J. Physiol. 589, 3983–3994. 10.1113/jphysiol.2011.213363 21690193 PMC3179997

[B4] Baggish A. L. Park J. Min P. K. Isaacs S. Parker B. A. Thompson P. D.. (2014). Rapid upregulation and clearance of distinct circulating microRNAs after prolonged aerobic exercise. J. Appl. Physiol. 116, 522–531. 10.1152/japplphysiol.01141.2013 24436293 PMC3949215

[B5] Berthoin S. Gerbeaux M. Turpin E. Guerrin F. Lensel-Corbeil G. Vandendorpe F. (1994). Comparison of two field tests to estimate maximum aerobic speed. J. Sports Sci. 12, 355–362. 10.1080/02640419408732181 7932945

[B6] Borg G. A. (1982). Psychophysical bases of perceived exertion. Med. Sci. Sports Exerc. 14, 377–381. 10.1249/00005768-198205000-00012 7154893

[B7] Burgomaster K. A. Howarth K. R. Phillips S. M. Rakobowchuk M. Macdonald M. J. McGee S. L. (2008). Similar metabolic adaptations during exercise after low volume sprint interval and traditional endurance training in humans. J. Physiol. 586, 151–160. 10.1113/jphysiol.2007.142109 17991697 PMC2375551

[B8] Chen X. Liang H. Zhang J. Zen K. Zhang C. Y. (2012). Secreted microRNAs: a new form of intercellular communication. Trends Cell Biol. 2, 2125–2132. 10.1016/j.tcb.2011.12.001 22260888

[B9] Costigan S. A. Eather N. Plotnikoff R. C. Taaffe D. R. Lubans D. R. (2015). High-intensity interval training for improving health-related fitness in adolescents: a systematic review and meta-analysis. Br. J. Sports Med. 49, 1253–1261. 10.1136/bjsports-2014-094490 26089322

[B10] Da Silva N. D. Jr. Fernandes T. Soci U. P. Monteiro A. W. Phillips M. I. DE Oliveira E. M. (2012). Swimming training in rats increases cardiac MicroRNA-126 expression and angiogenesis. Med. Sci. Sports Exerc. 44, 1453–1462. 10.1249/MSS.0b013e31824e8a36 22330028

[B11] Deregibus M. C. Cantaluppi V. Calogero R. Lo Iacono M. Tetta C. Biancone L.. (2007). Endothelial progenitor cell derived microvesicles activate an angiogenic program in endothelial cells by a horizontal transfer of mRNA. Blood 110, 2440–2448. 10.1182/blood-2007-03-078709 17536014

[B12] Dickhuth H. H. Yin L. Niess A. Röcker K. Mayer F. Heitkamp H. C.. (1999). Ventilatory, lactate-derived and catecholamine thresholds during incremental treadmill running: relationship and reproducibility. Int. J. Sports Med. 20, 122–127. 10.1055/s-2007-971105 10190774

[B13] Eskelinen J. J. Heinonen I. Löyttyniemi E. Hakala J. Heiskanen M. A. Motiani K. K.. (2016). Left ventricular vascular and metabolic adaptations to high-intensity interval and moderate intensity continuous training: a randomized trial in healthy middle-aged men. J. Physiol. 594, 7127–7140. 10.1113/JP273089 27500951 PMC5134384

[B14] Eskelinen J. J. Heinonen I. Löyttyniemi E. Saunavaara V. Kirjavainen A. Virtanen K. A.. (2015). Muscle-specific glucose and free fatty acid uptake after sprint interval and moderate-intensity training in healthy middle-aged men. J. Appl. Physiol. 118, 1172–1180. 10.1152/japplphysiol.01122.2014 25767035

[B15] Fernandes T. Magalhães F. C. Roque F. R. Phillips M. I. Oliveira E. M. (2012). Exercise training prevents the microvascular rarefaction in hypertension balancing angiogenic and apoptotic factors: role of microRNAs-16, -21, and -126. Hypertension 59, 513–520. 10.1161/HYPERTENSIONAHA.111.185801 22215713

[B16] Fichtlscherer S. De Rosa S. Fox H. Schwietz T. Fischer A. Liebetrau C.. (2010). Circulating microRNAs in patients with coronary artery disease. Circ. Res. 107, 677–684. 10.1161/CIRCRESAHA.109.215566 20595655

[B17] Filipowicz W. Bhattacharyya S. N. Sonenberg N. (2008). Mechanisms of post-transcriptional regulation by microRNAs: are the answers in sight? Nat. Rev. Genet. 9, 102–114. 10.1038/nrg2290 18197166

[B18] Flowers E. Won G. Y. Fukuoka Y. (2015). MicroRNAs associated with exercise and diet: a systematic review. Physiol. Genomics 47, 1–11. 10.1152/physiolgenomics.00095.2014 25465031 PMC7199230

[B19] Harris T. A. Yamakuchi M. Kondo M. Oettgen P. Lowenstein C. J. (2010). Ets-1 and Ets-2 regulate the expression of microRNA-126 in endothelial cells. Arterioscler. Thromb. Vasc. Biol. 30, 1990–1997. 10.1161/ATVBAHA.110.211706 20671229 PMC3121560

[B20] Haskell W. L. Lee I. M. Pate R. R. Powell K. E. Blair S. N. Franklin B. A.. (2007). Physical activity and public health: updated recommendation for adults from the American College of Sports Medicine and the American Heart Association. Circulation 116, 1081–1093. 10.1161/CIRCULATIONAHA.107.185649 17671237

[B21] Hergenreider E. Heydt S. Tréguer K. Boettger T. Horrevoets A. J. Zeiher A. M.. (2012). Atheroprotective communication between endothelial cells and smooth muscle cells through miRNAs. Nat. Cell Biol. 14, 249–256. 10.1038/ncb2441 22327366

[B22] Huntzinger E. Izaurralde E. (2011). Gene silencing by microRNAs: contributions of translational repression and mRNA decay. Nat. Rev. Genet. 12, 99–110. 10.1038/nrg2936 21245828

[B23] Jansen F. Yang X. Proebsting S. Hoelscher M. Przybilla D. Baumann K.. (2014). MicroRNA expression in circulating microvesicles predicts cardiovascular events in patients with coronary artery disease. J. Am. Heart Assoc. 3:e001249. 10.1161/JAHA.114.001249 25349183 PMC4338711

[B24] Jung H. J. Suh Y. (2014). Circulating miRNAs in ageing and ageing-related diseases. J. Genet. Genomics 41, 465–472. 10.1016/j.jgg.2014.07.003 25269672 PMC4354804

[B25] Kilian Y. Wehmeier U. F. Wahl P. Mester J. Hilberg T. Sperlich B. (2016). Acute response of circulating vascular regulating microRNAs during and after high-intensity and high-volume cycling in children. Front. Physiol. 7:92. 10.3389/fphys.2016.00092 27014090 PMC4789462

[B26] Léger L. Boucher R. (1980). An indirect continuous running multistage field test: the Université de Montréal track test. Can. J. Appl. Sport Sci. 5, 77–84. 7389053

[B27] Makarova J. A. Maltseva D. V. Galatenko V. V. Abbasi A. Maximenko D. G. Grigoriev A. I.. (2014). Exercise immunology meets MiRNAs. Exerc. Immunol. Rev. 20, 135–164. 24974725

[B28] McAlexander M. A. Phillips M. J. Witwer K. W. (2013). Comparison of methods for miRNA extraction from plasma and quantitative recovery of RNA from cerebrospinal fluid. Front. Genet. 4:83. 10.3389/fgene.2013.00083 23720669 PMC3655275

[B29] McCarthy J. J. Esser K. A. (2007). MicroRNA-1 and microRNA-133a expression are decreased during skeletal muscle hypertrophy. J. Appl. Physiol. 102, 306–313. 10.1152/japplphysiol.00932.2006 17008435

[B30] McGehee J. C. Tanner C. J. Houmard J. A. (2005). A comparison of methods for estimating the lactate threshold. J. Strength Cond. Res. 19, 553–558. 10.1519/15444.1 16095403

[B31] Milanović Z. Sporiš G. Weston M. (2015). Effectiveness of High-Intensity Interval Training (HIT) and continuous endurance training for VO2max improvements: a systematic review and meta-analysis of controlled trials. Sports Med. 45, 1469–1481. 10.1007/s40279-015-0365-0 26243014

[B32] Myers J. Prakash M. Froelicher V. Do D. Partington S. Atwood J. E. (2002). Exercise capacity and mortality among men referred for exercise testing. N.Engl. J. Med. 346, 793–801. 10.1056/NEJMoa011858 11893790

[B33] Oikawa M. Abe M. Kurosawa H. Hida W. Shirato K. Sato Y. (2001). Hypoxia induces transcription factor ETS-1 via the activity of hypoxia-inducible factor-1. Biochem. Biophys. Res. Commun. 289, 39–43. 10.1006/bbrc.2001.5927 11708773

[B34] Poissonnier L. Villain G. Soncin F. Mattot V. (2014). miR126-5p repression of ALCAM and SetD5 in endothelial cells regulates leucocyte adhesion and transmigration. Cardiovasc. Res. 102, 436–447. 10.1093/cvr/cvu040 24562769

[B35] Polakovičová M. Musil P. Laczo E. Hamar D. Kyselovič J. (2016). Circulating microRNAs as potential biomarkers of exercise response. Int. J. Mol. Sci. 17:E1553. 10.3390/ijms17101553 27782053 PMC5085619

[B36] Radom-Aizik S. Zaldivar F. Jr Oliver S. Galassetti P. Cooper D. M. (2010). Evidence for microRNA involvement in exercise-associated neutrophil gene expression changes. J. Appl. Physiol. 109, 252–261. 10.1152/japplphysiol.01291.2009 20110541 PMC2904199

[B37] Roecker K. Schotte O. Niess A. M. Horstmann T. Dickhuth H. H. (1998). Predicting competition performance in long-distance running by means of a treadmill test. Med. Sci. Sports Exerc. 30, 1552–1557. 10.1097/00005768-199810000-00014 9789858

[B38] Sayed D. Abdellatif M. (2011). MicroRNAs in development and disease. Physiol. Rev. 91, 827–887. 10.1152/physrev.00006.2010 21742789

[B39] Schlosser K. McIntyre L. A. White R. J. Stewart D. J. (2015). Customized internal reference controls for improved assessment of circulating microRNAs in disease. PLoS ONE 10:e0127443. 10.1371/journal.pone.0127443 26010841 PMC4444297

[B40] Schmitz B. Klose A. Schelleckes K. Jekat C. M. Krüger M. Brand S. M. (2017). Yo-Yo IR1 vs incremental continuous running test for prediction of 3000 m performance. J. Sports Med. Phys. Fitness. 10.23736/S0022-4707.17.07097-9. [Epub ahead of print]. 28229568

[B41] Schober A. Nazari-Jahantigh M. Wei Y. Bidzhekov K. Gremse F. Grommes J.. (2014). MicroRNA-126-5p promotes endothelial proliferation and limits atherosclerosis by suppressing Dlk1. Nat. Med. 20, 368–376. 10.1038/nm.3487 24584117 PMC4398028

[B42] Simionescu-Bankston A. Kumar A. (2016). Noncoding RNAs in the regulation of skeletal muscle biology in health and disease. J. Mol. Med. 94, 853–866. 10.1007/s00109-016-1443-y 27377406 PMC4957971

[B43] Sonveaux P. Copetti T. De Saedeleer C. J. Végran F. Verrax J. Kennedy K. M.. (2012). Targeting the lactate transporter MCT1 in endothelial cells inhibits lactate-induced HIF-1 activation and tumor angiogenesis. PLoS ONE 7:e33418. 10.1371/journal.pone.0033418 22428047 PMC3302812

[B44] Uhlemann M. Möbius-Winkler S. Fikenzer S. Adam J. Redlich M. Möhlenkamp S.. (2014). Circulating microRNA-126 increases after different forms of endurance exercise in healthy adults. Eur. J. Prev. Cardiol. 21, 484–491. 10.1177/2047487312467902 23150891

[B45] Wang S. Aurora A. B. Johnson B. A. Qi X. McAnally J. Hill J. A.. (2008). The endothelial-specific microRNA miR-126 governs vascular integrity and angiogenesis. Dev. Cell 15, 261–271. 10.1016/j.devcel.2008.07.002 18694565 PMC2685763

[B46] Wehmeier U. F. Hilberg T. (2014). Capillary earlobe blood may be used for RNA isolation, gene expression assays and microRNA quantification. Mol. Med. Rep. 9, 211–216. 10.3892/mmr.2013.1779 24213018

[B47] Weston K. S. Wisløff U. Coombes J. S. (2014). High-intensity interval training in patients with lifestyle-induced cardiometabolic disease: a systematic review and meta-analysis. Br. J. Sports Med. 48, 1227–1234. 10.1136/bjsports-2013-092576 24144531

[B48] Wu X. D. Zeng K. Liu W. L. Gao Y. G. Gong C. S. Zhang C. X.. (2014). Effect of aerobic exercise on miRNA-TLR4 signaling in atherosclerosis. Int. J. Sports Med. 35, 344–350. 10.1055/s-0033-1349075 24022569

[B49] Zacharewicz E. Lamon S. Russell A. P. (2013). MicroRNAs in skeletal muscle and their regulation with exercise, ageing, and disease. Front. Physiol. 4:266. 10.3389/fphys.2013.00266 24137130 PMC3786223

[B50] Zampetaki A. Kiechl S. Drozdov I. Willeit P. Mayr U. Prokopi M.. (2010). Plasma microRNA profiling reveals loss of endothelial miR-126 and other microRNAs in type 2 diabetes. Circ. Res. 107, 810–817. 10.1161/CIRCRESAHA.110.226357 20651284

[B51] Zernecke A. Bidzhekov K. Noels H. Shagdarsuren E. Gan L. Denecke B.. (2009). Delivery of microRNA-126 by apoptotic bodies induces CXCL12-dependent vascular protection. Sci. Signal. 2:ra81. 10.1126/scisignal.2000610 19996457

[B52] Zhang J. Li S. Li L. Li M. Guo C. Yao J.. (2015). Exosome and exosomal microRNA: trafficking, sorting, and function. Genomics Proteomics Bioinform. 13, 17–24. 10.1016/j.gpb.2015.02.001 25724326 PMC4411500

