# Supplementary material for: Dose-Response of High-Intensity Training (HIT) on Atheroprotective miRNA-126 Levels
Source: Front Physiol. 2017 May 30;8:349. doi: 10.3389/fphys.2017.00349 (PMC5447767; doi:10.3389/fphys.2017.00349)
Supplement: Supplementary file 1 [file DataSheet1.PDF]

Supplemental Material

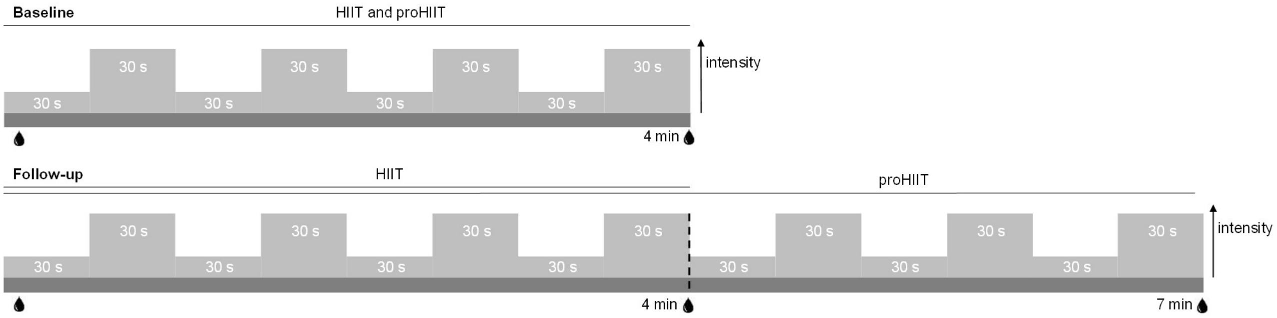

Supplemental figure 1:

Schematic representation of high-intensity runs and blood sampling at baseline and follow-up. At baseline, the high-intensity interval training (HIIT) group and the progressive high-intensity interval training (proHIIT) group performed an identical exercise protocol with high-intensity runs at maximum speed for 30 s (all-out) with 30 s of active recovery periods at warm-up speed. Blood sampling was performed immediately before and after exercise (4 min) for determination of miR-126-3p/ -5p and lactate concentration. After the intervention, the proHIIT group performed three additional exercise bouts with blood sampling during the exercise at 4 min and after the exercise at 7 min. Blood sampling after intervention in the HIIT group was performed after the exercise at 4 min and after 3 min of rest (7 min after start of the protocol).

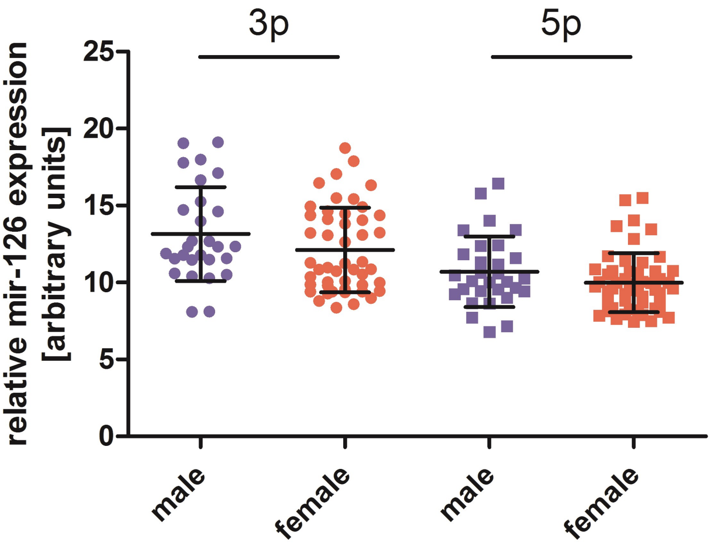

Supplemental figure 2:

Comparison of female and male microRNA-126-3p and -5p levels at baseline. No significant difference was detected in dependence of sex.

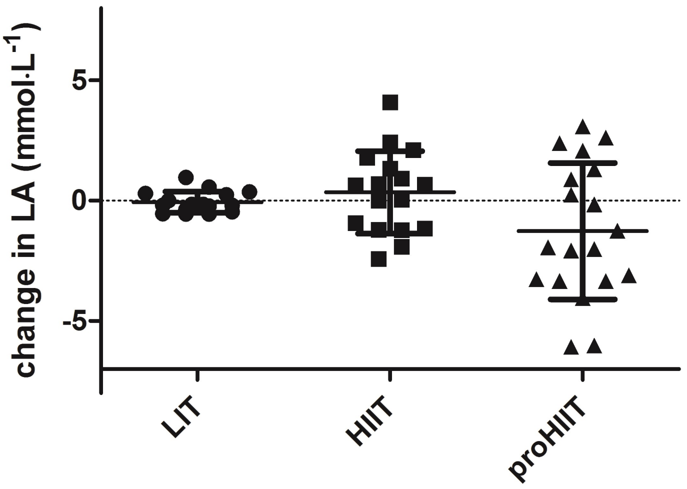

Supplemental figure 3:

Change in post-exercise blood lactate (LA) concentration in dependence of training groups. Exercise LA was significantly reduced in the progressive high-intensity interval training (proHIIT, $p = 0.0488$, pre- *vs* post-intervention) group. No difference was detected in the low-intensity training (LIT) or high-intensity interval training (HIIT) group.
